# Profiling the alterations of serum proteome in dairy cows with retained placenta using high-throughput tandem mass tags quantitative approach

**DOI:** 10.1080/01652176.2023.2164908

**Published:** 2023-01-13

**Authors:** Anđelo Beletić, Josipa Kuleš, Dina Rešetar Maslov, Vladimir Farkaš, Ivana Rubić, Blanka Beer Ljubić, Dražen Đuričić, Damir Žubčić, Marko Samardžija, Vladimir Mrljak

**Affiliations:** a Laboratory of proteomics, Internal Diseases Clinic, Faculty of Veterinary Medicine, University of Zagreb, Zagreb, Croatia; b Department of Chemistry and Biochemistry, Faculty of Veterinary Medicine, University of Zagreb, Zagreb, Croatia; c Internal Diseases Clinic, Faculty of Veterinary Medicine, University of Zagreb, Zagreb, Croatia; d Mount-Trade D.O.O, Garešnica, Croatia; e Reproduction and Obstetrics Clinic, Faculty of Veterinary Medicine, University of Zagreb, Zagreb, Croatia

**Keywords:** Cow, bovine, dairy, retained placenta, serum, proteomics, lipopolysaccharide-binding protein

## Abstract

**Background:**

Retained placenta (RP), a quite common disorder in dairy cows, shows a high negative impact on their health status and milk production.

**Aim:**

To investigate the difference in the serum proteome between the cows with RP and the physiologic puerperium (PP).

**Material & Methods:**

Analysis of serum samples from nine cows with RP and six with PP using high-resolution liquid chromatography-tandem mass spectrometry approach. The proteins differing in the relative abundance between the PP and RP groups were classified using the Protein Analysis Through Evolutionary Relationship tool. For the pathway enrichment analysis, the REACTOME tool, with the human genome as the background, was employed. The criterion for significance was the false discovery rate corrected P-value less than 0.05.

**Results:**

In total 651 proteins were identified with altered relative abundance of ten proteins. Among them, seven had higher, and three showed lower relative abundance in RP than in the PP group. The differently abundant proteins participated in 15 pathways: six related to hemostasis, three involved in lipoprotein metabolism, and the remaining ones associated with for instance redox homeostasis, post-translational modification, and scavenging. Finally, the validation of the proteomic results showed that haptoglobin and lipopolysaccharide-binding protein levels reliably differentiated between the RP and PP groups.

**Conclusion:**

The pattern of serum proteome alterations in the cows with RP mirrored several interplaying mechanisms underlying the systematic response to the presence of RP, therefore representing a source to mine for predictive or prognostic biomarkers.

## Graphical Abstract

**Figure:**
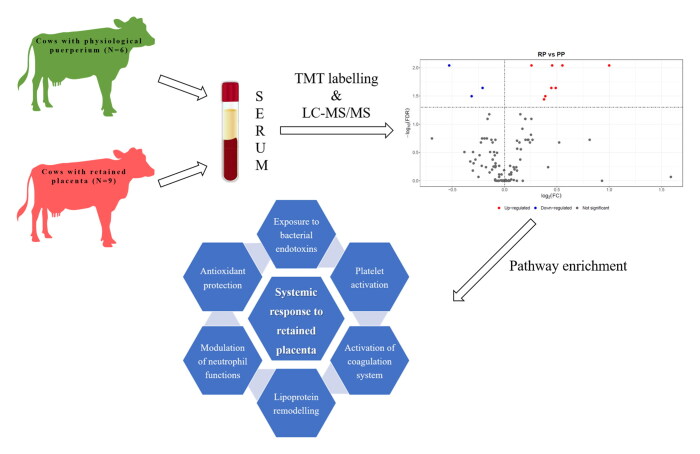

## 1. Introduction

Retained placenta (RP) or retained fetal membranes in the dairy cows is a pathologic syndrome occurring when a cow does not achieve to completely expel the fetal membranes within 12–24 h after the calving (Peter 2013; Sheldon 2018). It represents a frequent health problem, as illustrated by the average incidence ranging from 4 to 16%. Prompt therapeutic actions are necessary to reduce the deleterious interactions between the endometrium and the RP tissue. However, despite the improvements in the management protocols, the link is persisting between RP and a comprehensive plethora of health and economic issues such as uterine and abdominal infections, increased costs of veterinary service, decreased milk yield, lowered fertility, and culling (Dervishi and Ametaj 2017; Maletić et al. 2022).

The preceding eight decades of research established an extensive list of RP pathological features and risk factors, which is still far from complete (Peter 2013). The most important immune-metabolic components in RP pathogenesis represent loosely regulated periparturient inflammation, altered neutrophil functionality, ineffective glucose consumption, overwhelmed lipid mobilization, hypocalcemia, hyposelenemia, and vitamin E deficiency (Dervishi and Ametaj 2017; Le Blanc, 2020; Horst et al. 2021). The presence of the bacterial endotoxins additionally increases the probability of RP occurrence. The obstetric factors, like dystocia, uterine torsions, and cesareans, antepartum immune hyperreactivity, and placentomes-related pathology can also directly participate in RP pathogenesis. Stress, calving season, sex, and fetus anomalies are the additional factors that contribute to the RP risk in an indirect manner (Peter 2013). Also, the likelihood of RP can be higher due to the genetic predisposition (Dervishi and Ametaj 2017).

In the previous proteomic studies of RP, the researchers have focused on the tissue proteome. Using the gel-based proteomic approach they showed differences in the tissue proteome composition between the retained and normally expelled placentas (Fu et al. 2016, Wawrzykowski et al. 2019). In the maternal part of the placentome, Wawrzykowski et al. (2019) described the proteome changes which involved seven proteins, all of them being more abundant in the RP tissue. Among them, the highest increase had ferritin heavy chain, a protein relevant for the cell proliferation processes, being almost six times more abundant in the retained tissue. In the same study (Wawrzykowski et al. 2019) nineteen proteins showed alterations in the fetal part of the placentome, whereby an approximately equal number of them had increased and decreased abundance in the retained tissue compared to the physiologically expelled. The lowest relative abundance had the mitochondrial persulfide dioxygenase ETHE1, a cell redox homeostasis enzyme, with the amount eleven times less in the RP than in the physiologically expelled placenta. On the other hand, the highest relative abundance, exceeding the value of nine, was present for peptidyl-prolyl cis-trans isomerase A, an enzyme participating in protein folding.

Therefore, a hypothesis emerged that the composition of cows’ serum proteome could also change in the presence of RP. In line with that, the principal aim of the study was to compare the sera of the cows with RP and the physiological puerperium (PP) regarding the abundance of the proteins identified using high-throughput tandem mass tag-based liquid chromatography-tandem mass spectrometry approach. In addition, the objective was to assess the relationship between the alterations in the proteome composition and the intensity of the acute phase reaction (APR), glycemia, calcemia, and lipolysis indicators.

## 2. Material and methods

### 2.1. Animals and blood sampling

The study included 15 cows, nine with RP and six with PP, from private farms in the Zagreb County, Croatia. The cows were kept in separate barns. They were all of the Simmental breed, except one, being crossbreed with Holstein Friesian breed. The cows were multiparous, with the age ranging between three and five years. All cows had spontaneous calving. Non-specific clinical signs, such as agitation, inappetence, tachycardia, and tachypnoea, appeared in cows with RP soon after the delivery. Every cow underwent a gynecological examination 12 − 24 h after the calving, and RP was diagnosed if the fetal membranes were seen hanging from the vulva or remained inside the uterus (Djuricic et al. 2011; Peter 2013; Sheldon 2018). The treatment included the application of 1-2 g of oxytetracycline (Geomycin F® tablets) between the uterus and placenta. The dose of 1 g of oxytetracycline had been repeated every 48 h for ten days (Djuricic et al. 2011). Three cows with RP developed signs of systemic illness: body temperature reaching 41 °C, pronounced tachycardia, tachypnoea, and decreased ruminations. These cows received a single dose of ceftiofur (1 mg/kg of body weight, *i.m.*) alongside the first dose of oxytetracycline. All cows recovered successfully.

In both groups, blood samples were collected from *V. coccygea*, during the first visit, 12 − 24 h after the calving. After clotting and centrifugation at 1500 x *g* at 4 °C for 15 min, serum was separated into Eppendorf tubes, and stored it at −80 °C until analysis.

The Ethical Committee of the Faculty of Veterinary Medicine, University of Zagreb, Croatia, approved the study (Class: 640-01/14-17/51; Registry number: 251-61-01/139-14-1).

### 2.2. Sample preparation and LC-MS/MS analysis

The protocol as published by Horvatić et al. (2019) was followed to prepare the serum samples. First, total protein concentration in a sample was measured with the Pierce BCA Protein Assay Kit (Thermo Scientific, Rockford, IL, USA). The sample volume, containing 35 μg of proteins, was diluted to 50 μL with 0.1 M triethylammonium bicarbonate (TEAB, Thermo Scientific, USA). The next step included protein reduction with 2.5 μL of 200 mM dithiothreitol (Sigma Aldrich, St. Louis, MO, USA) for 60 min at 55 °C, alkylation with 2.5 μL of 375 mM iodoacetamide (Sigma Aldrich, USA) for 30 min at 20 − 25 °C protected from the light, and overnight precipitation at −20 °C by adding 300 μL of acetone. After centrifugation at 8000 x g at 4 °C for 15 min, dissolving the protein pellets in 50 μL of 0.1 M TEAB, and adding 1 μL of 1 mg/mL trypsin solution (Promega, Madison, WI, USA), overnight digestion at 37 °C followed. In parallel, the internal standard was prepared as a mixture of an equivalent quantity of proteins from all samples, which was necessary as a reference during the quantitative analysis. For labelling, the individual samples or internal standard were mixed with the specific Tandem Mass Tag reagent (TMT sixplex™ Isobaric Label Reagent Set, Thermo Scientific, USA). Following incubation at room temperature and terminating the labelling reaction with 5% hydroxylamine (Sigma-Aldrich, USA), the internal standard and five samples were randomly combined.

For the high-resolution LC-MS/MS analysis of TMT-labelled peptides, the protocol from previous studies (Kuleš et al. 2021) was followed. The Ultimate 3000 RSLCnano instrument (Dionex, Germering, Germany) was used for the chromatographic separation. After being dissolved in the loading solution (2% acetonitrile (ACN), 0.1% formic acid), and concentrated and desalted on the trap column (C18 PepMap100, 5 μm, 100 A, 300 μ*m* × 5 mm; 12 min; flow rate of 15 μL/min), peptides were separated on the analytical column (PepMap™ RSLC C18, 50 cm × 75 μm) by combining two mobile phases: the mobile phase A, 0.1% formic acid in water, and the mobile phase B, 0.1% formic acid in 80% ACN. The separation protocol started with a linear gradient of 5–55% mobile phase B over 120 min, then continued with 55% to 95% for 1 min, held at 95% for 2 min, and returned at 5% B during 20 min at the flow rate of 300 nL/min. When separated, the peptides were ionized with a nanospray Flex ion source (Thermo Fisher Scientific, Bremen, Germany) containing a 10 μm-inner diameter SilicaTip emitter (New Objective, Littleton, MA, USA) and analyzed on Q Exactive Plus mass spectrometer (Thermo Fisher Scientific, Germany). The parameters for the mass spectrometry-based analysis were as follows: the positive ion mode using data-dependent analysis Top8 method; full scan MS spectra covering the m/z range from 350.0 to 1800.0 with a resolution of 70,000; injection time: 120 ms; AGC target: 1 × 10^6^; isolation window: ± 2.0 Da; dynamic exclusion: 30 s; HCD fragmentation at step collision energy (29% and 35% NCE) with a resolution of 17,500; AGC target: 2 × 10^5^; criteria for the precursor ions exclusion from fragmentation: the unassigned charge state, or charge states of +1 and more than +7. To perform qualitative and quantitative analysis from the obtained spectra, we applied the SEQUEST algorithm in the Proteome Discoverer software (v. 2.3., ThermoFisher Scientific, Bremen, Germany) and searched for the data against the UniProt *Bos taurus* FASTA files (Release No. 2021_04; released on Nov 17 2021; 120,378 sequences) according to the following parameters: two trypsin missed cleavage sites; precursor tolerance 10 ppm; fragment mass compliance ± 0.02 Da; carbamidomethyl (C) fixed peptide modification, oxidation (M), and TMT sixplex (K, peptide N-terminus) dynamic modifications. The false discovery rate (FDR) for peptide identification was computed according to the Percolator algorithm in the Proteome Discoverer workflow based on the search results against a decoy database and was set at 1% FDR. The criteria for protein identification were: the presence of at least two unique peptides and the 5% FDR. For the proteins’ quantification, the first step was to correlate the relative intensities of the extracted reporter ions and the fragmented peptides. After that, we calculated the proteins’ relative abundances as the ratio between the relative intensities in the individual sample and the internal standard of the corresponding TMT sixplex™.

The mass spectrometry proteomics data have been deposited to the ProteomeXchange Consortium *via* the PRIDE (Perez-Riverol et al. 2022) partner repository with the dataset identifier PXD034963, username: reviewer_pxd034963@ebi.ac.uk, and password V0Vmghzk.

### 2.3. Methodology for validation of the LC-MS/MS results

For validation purposes, the concentration of lipopolysaccharide-binding protein (LBP), fibrinogen alpha chain (FGA), and tetranectin (TNCT) was measured with the bovine-specific commercially available enzyme-linked immunosorbent assays (ELISA) kits according to the Manufacturerʼs instructions (Assay Genie, Dublin, Ireland). For the LBP kit (Bovine Lipopolysaccharide-binding protein (LBP) ELISA kit; Catalogue CodeBOEB0664), the Manufacturer declared sensitivity 0.281 ng/mL, intra-assay coefficient of variation (CV) 6.2%, inter-assay CV 8.5%, and spike recovery 97%. The specifications for the FGA kit (Bovine Fibrinogen alpha chain (FGA) ELISA kit; Catalogue CodeBOEB0567) were: sensitivity 3.0 ng/mL, intra-assay CV 6.2%, inter-assay CV 9.1%, and spike recovery 113%. The TNCT kit (Bovine Tetranectin (CLEC3B) ELISA kit; Catalogue CodeBOEB0666) had the following characteristics: sensitivity 0.31 ng/mL, intra-assay CV 6.5%, inter-assay CV less 9.1%, and spike recovery 106%. Haptoglobin (HP) concentration was measured with the previously validated *in house* developed spectrophotometric assay (Turk et al. 2021). The activity of glutathione peroxidase (GPX) was assessed with the commercial spectrophotometric kit (Randox Laboratories Ltd, Dublin, Ireland). The automated analyzer Abbott Architect c4000 (Abbott, Abbott Park, IL, USA) was used for the spectrophotometric assays.

### 2.4. Methodology for the routine biochemistry parameters

Commercially available kits (Abbott, USA; Randox Laboratories Ltd, Ireland; DiaSys GmbH, Holzheim, Germany) run on the automated analyzer Abbott Architect c4000 (Abbott, USA) were used to measure glucose, urea, creatinine, total protein, albumin (ALB), total bilirubin (T-BIL), cholesterol (CHOL), triglycerides (TRIG), non-esterified fatty acids (NEFA), β-hydroxybutyrate (BHB), aspartate aminotransferase (AST), gamma-glutamyl transferase, lactate dehydrogenase (LDH), and glutamate dehydrogenase. The concentration of serum amyloid A (SAA) was analyzed using the commercially available ELISA kit (Tridelta Development Ltd, Maynooth, Ireland) and indicated the intensity of APR.

### 2.5. Statistical analyses and bioinformatics

To analyze the difference in the relative abundance of proteins between the PP and RP groups, we used R software v.4.1.2. (http://www.r-project.org/.) (Accessed July 10 2021).), following the detailed instructions by Kuleš et al. (2021). The official gene symbols replaced the UniProtKB identifiers *via* the UniProtKB ID mapping tool (https://www.uniprot.org/uploadlists/), which allowed the Gene Ontology (GO) classification, using the Protein Analysis Through Evolutionary Relationship (PANTHER) tool (http://www.pantherdb.org/) with the subset of GO terms (GO Slim database) (Mi et al. 2021). The next step was pathway enrichment analysis with the REACTOME tool (https://reactome.org/), using the human genome as the background. The criterion to denote a pathway as significant was the FDR corrected P-value less than 0.05 (Gillespie et al. 2022). For the validation of results and the routine biochemistry tests, the statistical evaluation included the Mann-Whitney U test, Receiver Operating Characteristic (ROC) curve analysis, Spearman’s rank-order correlation, and multiple regression analysis, all performed in MedCalc® software (v.12.5.0.0), using the P-value below 0.05 as significant.

## 3. Results

### 3.1. Proteomics and bioinformatics analysis

**Figure 1.**
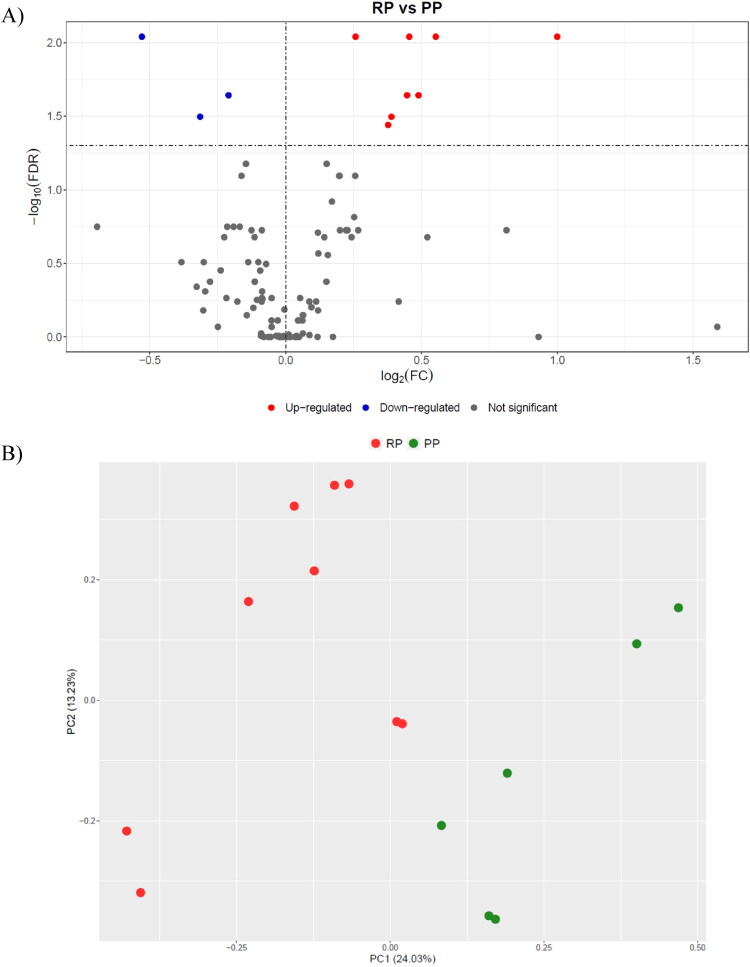
Statistical analysis of proteomics results: A) Volcano plot showing the differences in the abundance of proteins between the retained placenta (RP; *n* = 9) and the physiological puerperium (PP; *n* = 6) groups; B) Principal Component Analysis (PCA) score plot showing samples from cows with RP and PP.

The analysis of the MS/MS spectra identified 651 proteins in total (Table S1 in the Supplement), among which 114 were master proteins. The volcano plot in Figure 1A showed that eight master proteins were more abundant in the RP group. As shown in Table 1, this group of proteins, beside HP, GPX, LBP, inter-alpha-trypsin inhibitor heavy chain H4 (ITIH4), FGA, and hemopexin (HPX) contained two proteins, initially marked as uncharacterized, which were both finally characterized as immunoglobulin heavy constant mu (IGHM) after the BLAST analysis. Also, three master proteins had a lower abundance in the RP group: tetranectin, serpin family D member 1, and apolipoprotein A4. Finally, the principal component analysis score plot (Figure 1B) indicated acceptable discrimination between the RP and PP groups. Further, Table 1 presents also the details about the proteins with different relative abundance and the corresponding statistical features.

**Table 1.** The proteins with the different relative abundance (ratio between the amount in an individual sample and internal standard) in the cows with the retained placenta (RP; *n* = 9) and those with the physiological puerperium (PP; *n* = 6).

			Abundance				
			median (min − max)				
Accession	Gene	Description	RP (*n* = 9)	PP (*n* = 6)	FC	P − value	FDR
*Higher abundance in the RP group*							
Q2TBU0	HP	Haptoglobin	1.10 (0.95 − 1.67)	0.59 (0.49 − 0.80)	1.86	<0.001	0.010
A0A4W2I9H0	GPX3	Glutathione peroxidase	1.22 (0.92 − 1.51)	0.86 (0.75 − 0.96)	1.42	0.002	0.026
A0A4W2HPU3	BPI	Lipopolysaccharide-binding protein	1.20 (1.05 − 1.48)	0.85 (0.75 − 0.89)	1.41	<0.001	0.010
Q3T052	ITIH4	Inter-alpha-trypsin inhibitor heavy chain H4	1.07 (0.93 − 1.39)	0.79 (0.55 − 0.97)	1.35	0.002	0.023
A0A3Q1M032	IGHM	Uncharacterized protein*	1.01 (0.93 − 1.49)	0.81 (0.74 − 0.88)	1.25	<0.001	0.009
A0A4W2H221	FGA	Fibrinogen alpha chain	1.08 (0.91 − 1.51)	0.88 (0.84 − 1.38)	1.22	0.003	0.042
A0A4W2EKG5	HPX	Hemopexin	1.10 (1.01 − 1.20)	0.92 (0.86 − 0.97)	1.20	0.004	0.010
G5E5T5	IGHM	Uncharacterized protein*	1.01 (0.86 − 1.49)	0.84 (0.79 − 0.92)	1.20	0.003	0.032
*Lower abundance in the RP group*							
Q2KIS7	CLEC3B	Tetranectin	1.01 (0.76 − 1.37)	1.13 (0.803 − 1.25)	0.89	0.003	0.038
A0A4W2G034	SERPIND1	Serpin family D member 1	0.93 (0.90 − 1.08)	1.11 (1.03 − 1.18)	0.84	0.002	0.023
A0A4W2CCP9	APOA4	Apolipoprotein A4	0.80 (0.69 − 0.99)	1.16 (1.07 − 1.35)	0.69	0.004	0.010

FC– Median fold change between RP and PP groups, calculated as the ratio of median in RP group to median in PP group.

FDR– false discovery rate.

*−The BLAST analysis characterized the protein as Immunoglobulin heavy constant mu (Accession: A0A4W2DVR2; Gene: IGHM).

**Figure 2.**
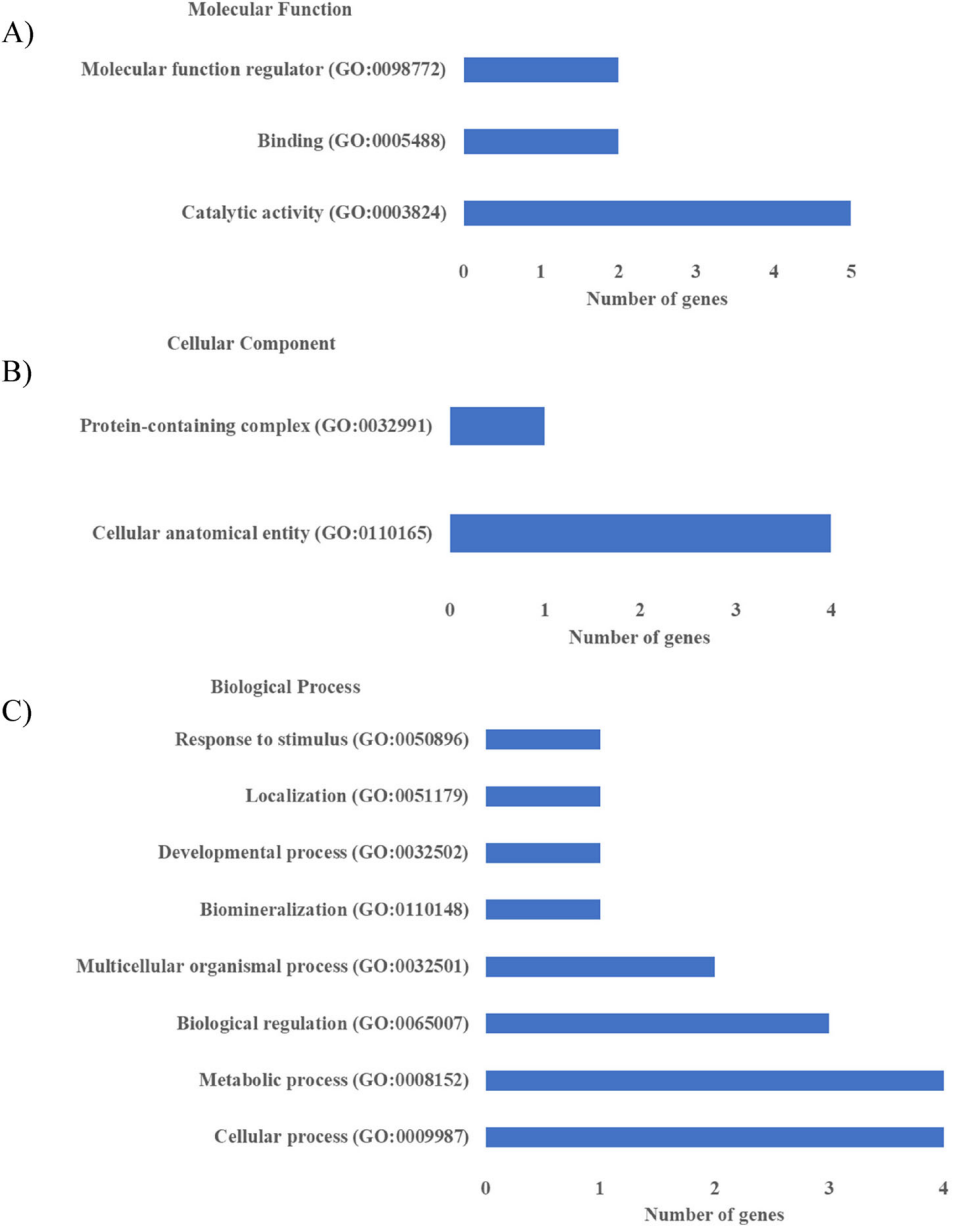
Gene Ontology for the proteins with the different abundance between the RP (*n* = 9) and PP (*n* = 6) groups using the PANTHER GO-Slim analysis: A) Molecular function; B) Cellular Component; C) Biological Process.

The GO classification of the proteins with the different abundance between the studied groups showed that the molecular function for more than half of them was catalytic (Figure 2A), and almost all of them were part of the cellular anatomic entity (Figure 2B). When classified by the biological process, cellular and metabolic processes had the highest number of the allocated genes (Figure 2C).

Also, proteins showing the difference in abundance between RP and PP groups were members of five protein classes: protein modifying enzymes (PC00260; two proteins), protein-binding activity modulators (PC00095; two proteins), intercellular signal molecule (PC00207; one protein), metabolite interconversion enzyme (PC00262; one protein) and transfer/carrier proteins (PC00219; one protein). Finally, the results from the REACTOME tool (Table 2) indicated that the differently abundant proteins participated in 15 pathways, of which six were related to hemostasis in global or specific compartments like platelet activation or fibrin clot formation.

**Table 2.** Pathway enrichment analysis of the proteins differentially abundant between the group with retained placenta (RP; *n* = 9) and the group with the physiological puerperium (PP; *n* = 6). FDR < 0.05 is considered significant.

Pathway	FDR	Count		Genes
		Observed	Background	
Platelet degranulation	<0.001	3	141	FGA, ITIH4, CLEC3B
Response to elevated platelet cytosolic Ca^2+^	<0.001	3	148	FGA, ITIH4, CLEC3B
Hemostasis	<0.001	5	803	FGA, IGHM, ITIH4, CLEC3B, SERPIND1
Platelet activation, signaling and aggregation	0.002	3	293	FGA, ITIH4, CLEC3B
Common Pathway of Fibrin Clot Formation	0.003	2	25	FGA, SERPIND1
Assembly of active LPL and LIPC lipase complexes	0.004	1	30	APOA4
Formation of Fibrin Clot (Clotting Cascade)	0.007	2	43	FGA, SERPIND1
Plasma lipoprotein remodeling	0.010	1	56	APOA4
Detoxification of Reactive Oxygen Species	0.014	1	69	GPX3
Amyloid fiber formation	0.021	2	89	FGA, APOA4
Plasma lipoprotein assembly, remodeling, and clearance	0.022	1	98	APOA4
Scavenging of heme from plasma	0.022	2	106	HPX, HP
Post-translational protein phosphorylation	0.023	2	109	FGA, SERPIND1
Regulation of IGF transport and uptake by IGFBP	0.026	2	127	FGA, SERPIND1
Binding and Uptake of Ligands by Scavenger Receptors	0.045	2	168	HPX, HP

FDR–False discovery rate (FDR) P–value; Insulin-like Growth Factor–IGF; Insulin-like Growth Factor Binding Proteins–IGFBP; Observed count–The number of mapped identifiers that match the pathway for the selected molecular type; Background count–The total number of identifiers in the pathway for the selected molecular type.

### 3.2. Validation of the LC-MS/MS results

Table 3 contains the results of LC-MS/MS data validation. The cows with RP had higher HP and LBP concentrations than those from the PP group, concordant with the proteomic data. Nevertheless, the analogous differences were absent for FGA, TNCT, and GPX (Table 3), which all had similar values in the studied groups. Further, cut-off for HP (0.19 g/L) and LBP (0.76 µg/mL) had identical sensitivity (95% Confidence Interval) of 100 (66–100) % and specificity of 100 (54–100) % in distinguishing the RP from PP group.

**Table 3.** Validation results: protein, results (median and minimum–maximum range) in cows with retention of the placenta (RP; *n* = 9) and cows with the physiologic puerperium (PP; *n* = 6), and P–values indicating the significance of differences between the groups (Mann-Whitney U test, P–value < 0.05 is considered significant).

Protein (unit)	RP (*n* = 9)	PP (*n* = 6)	P‒value
HP (g/L)	0.46 (0.36‒1.33)	0.05 (0.05‒0.18)	0.001
LBP (µg/mL)	4.16 (0.86‒18.76)	0.63 (0.44‒0.75)	0.002
FGA (ng/mL)	8.6 (1.3‒266.8)	27.4 (8.8‒378.3)	0.195
GPX (U/L)	579 (448‒970)	516 (291‒798)	0.289
TNCT (ng/mL)	1.5 (0.5‒24.9)	3.7 (1.0‒47.2)	0.409

Abbreviations: FGA– fibrinogen alpha chain; GPX–glutathione peroxidase; HP–haptoglobin; LBP–lipopolysaccharide-binding protein; TNCT– tetranectin.

### 3.3. Routine biochemistry parameters

The median SAA level was twenty times higher in the RP than in the PP group, indicating that a strong APR accompanied RP; glycemia and total bilirubin concentration were higher, and gamma-glutamyl transferase level lower in cows with RP (Table 4). Several parameters (ALB, urea, TRIG, CHOL, NEFA, BHB, and AST) had similar ratios in the studied groups, but their ratios were higher in the RP group (Table 4). Among the routine parameters only SAA showed satisfactory sensitivity and specificity to discriminate between the studied groups (Table 5).

**Table 4.** Biochemistry parameters/ratios: reference intervals (Laboratory of the Internal Diseases Clinic, Faculty of Veterinary Medicine, University of Zagreb), results (median and minimum–maximum range) in cows with retention of the placenta (RP; *n* = 9) and cows with the physiologic puerperium (PP; *n* = 6), and P–values indicating the significance of differences between the groups (Man-Whitney U test, *p* < 0.05 is considered significant).

Parameter/ratio (unit)	Reference interval	RP (*n* = 9)	PP (*n* = 6)	P‒value
SAA (mg/L)	< 2	667 (390‒1687)	33 (0.1‒69)	0.002
GLU (mmol/L)	2.5‒4.2	3.3 (1.0‒3.4)	2.2 (1.1‒2.8)	0.036
Urea (mmol/L)	4.1‒5.8	2.5 (1.3‒4.0)	1.3 (1.1‒3.3)	0.087
CREAT (μmol/L)	< 133	94 (78‒151)	105 (65‒142)	0.814
T-BIL (μmol/L)	2.1‒7.7	5.7 (3.9‒11.5)	2.5 (2.0‒4.4)	0.007
TP (g/L)	60‒80	70 (68‒77)	70 (68‒76)	0.813
ALB (g/L)	30‒36	27 (25‒33)	28 (26‒36)	0.287
ALB/Urea (g/mmol)	N/A	11 (8‒19)	22 (11‒24)	0.018
Calcium (mmol/L)	2.50‒2.90	2.28 (2.00‒2.39)	2.15 (2.02‒2.28)	0.596
Magnesium (mmol/L)	0.70‒1.00	0.85 (0.74‒1.25)	1.02 (0.85‒1.96)	0.088
Phosphate (mmol/L)	1.60‒2.30	1.61 (1.39‒1.95)	2.42 (0.87‒2.83)	0.059
CHOL (mmol/L)	2.3‒4.7	2.1 (1.1‒2.4)	2.3 (2.0‒5.3)	0.409
TRIG (mmol/L)	0.06‒0.22	0.17 (0.15‒0.22)	0.17 (0.11‒0.17)	0.214
NEFA (mmol/L)	< 0.70	0.82 (0.54‒1.65)	0.45 (0.17‒1.24)	0.077
(NEFA + TRIG)/CHOL	N/A	0.85 (0.30‒0.90)	0.28 (0.06‒0.62)	0.025
BHB (mmol/L)	< 1.40	0.86 (0.56‒2.37)	0.99 (0.76‒2.89)	0.195
AST (U/L)	< 123	102 (55‒165)	90 (54‒99)	0.077
AST/BHB (U/mmol)	N/A	108 (70‒148)	71 (31‒100)	0.025
AST/CHOL (U/mmol)	N/A	68 (23‒85)	44 (10‒45)	0.025
GGT (U/L)	< 25	20 (14‒29)	29 (22‒31)	0.032
GLDH (U/L)	< 30	11 (4‒14)	6 (7‒17)	0.289
CK (U/L)	< 94	235 (53‒346)	87 (82‒160)	0.224
LDH (U/L)	692‒1445	938 (640‒1298)	838 (730‒890)	0.050

Abbreviations: ALB–albumin; AST–aspartate aminotransferase; BHB–β-hydroxybutyrate; CHOL– cholesterol; CREAT–creatinine; GGT–gamma-glutamyl transferase; GLDH–glutamate dehydrogenase; GLOB–globulins; GLU–glucose; LDH–lactate dehydrogenase; N/A–not available; NEFA–non-esterified fatty acids; SAA–serum amyloid A; T-BIL–total bilirubin; TRIG–triglycerides; TP–total protein.

**Table 5.** Results of the ROC analysis for the routine biochemistry parameters with levels differing between cows with retention of the placenta (*n* = 9) and cows with the physiologic puerperium (*n* = 6): Area Under the Curve (AUC) with 95% Confidence Interval (CI), P-value, Cut-off, sensitivity (95% CI), and specificity (95% CI).

Parameter/ratio (unit)	AUC	P‒value	Cut-off	Sensitivity (%)	Specificity (%)
SAA (mg/L)	1.000 (0.782‒1.000)	<0.001	69	100 (66‒100)	100 (54‒100)
GLU (mmol/L)	0.861 (0.589‒0.982)	0.004	2.8	67 (30‒92)	100 (54‒100)
T-BIL (μmol/L)	0.926 (0.670‒0.997)	<0.001	4.4	78 (40‒97)	100 (54‒100)
ALB/Urea (g/mmol)	0.833 (0.556‒0.971)	0.001	19.2	100 (66‒100)	67 (22‒96)
(NEFA + TRIG)/CHOL	0.852 (0.578‒0.978)	0.002	0.29	100 (66‒100)	67 (22‒96)
AST/BHB (U/mmol)	0.852 (0.578‒0.978)	0.002	100	67 (30‒92)	100 (54‒100)
AST/CHOL (U/mmol)	0.852 (0.578‒0.978)	0.001	44	78 (40‒97)	100 (54‒100)
GGT (U/L)	0.833 (0.556‒0.971)	0.001	21	67 (30‒92)	100 (54‒100)

Abbreviations: ALB–albumin; AST–aspartate aminotransferase; BHB–β-hydroxybutyrate; CHOL– cholesterol; CREAT–creatinine; GGT–gamma-glutamyl transferase; GLDH–glutamate dehydrogenase; GLOB–globulins; GLU–glucose; LDH–lactate dehydrogenase; N/A–not available; NEFA–non-esterified fatty acids; SAA–serum amyloid A; T-BIL–total bilirubin; TRIG–triglycerides; TP–total protein.

### 3.4. Correlation between the extent of proteomic alterations and routine biochemistry parameters

**Figure 3.**
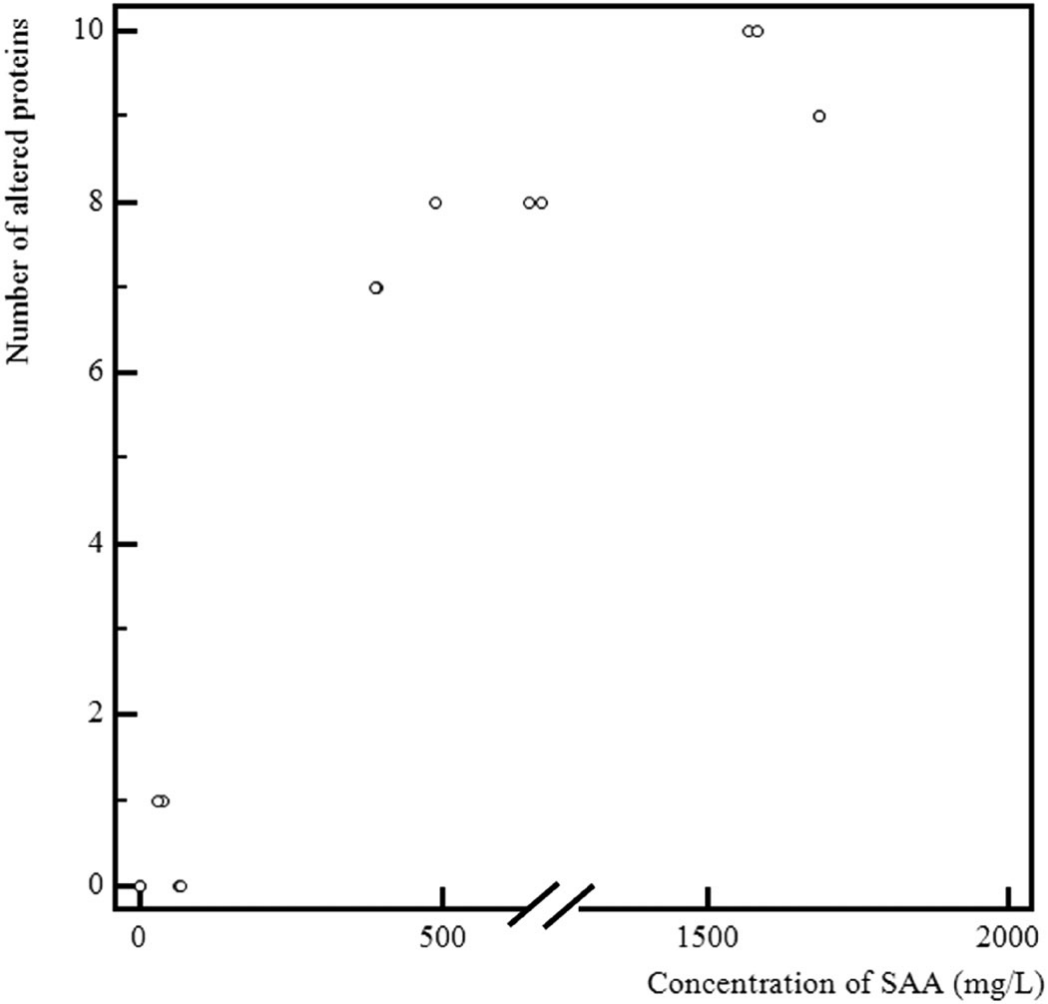
A graphical representation of the relationship between the number of altered proteins and serum amyloid A (SAA) concentration in serum.

The first step in assessing the extent of the proteomic alterations was to calculate the relative abundance median in all samples for each protein identified as having a different abundance between the study groups. We counted afterward how many proteins in every sample actually had the altered relative abundance, meaning higher than the median for the proteins with the RP-associated increase or lower for the proteins whose relative abundance decreased in RP. The alterations were found in only two cows with PP, each having one protein altered. In the RP group, the alterations occurred in the samples from all cows. The number of proteins having the altered abundance was between 7 and 10. Table 6 gives the detailed results for the correlation between the extent of the proteomic changes and the routine biochemistry parameters; a positive correlation existed with concentrations of SAA, T-Bil, TRIG, and NEFA, and the ratio (NEFA + TRIG)/CHOL; ALB was the only parameter showing a negative correlation. Multiple regression analysis identified only the relationship with SAA as significant (r_partial_=0.845, *p* < 0.001). Figure 3 shows a graphical representation of the relationship between the number of altered proteins and SAA concentration (in serum).

**Table 6.** Correlation between the number of proteins with the altered abundance and the routine biochemistry parameters: Spearman’s rho rank correlation coefficient (ρ) with the 95% confidence interval and P–value.

Parameter/ratio (unit)	ρ	P‒value
SAA (mg/L)	0.913 (0.752 to 0.971)	<0.001
GLU (mmol/L)	0.359 (-0.187 to 0.736)	0.188
Urea (mmol/L)	0.460 (-0.069 to 0.787)	0.084
CREAT (μmol/L)	−0.189 (-0.639 to 0.358)	0.500
T-BIL (μmol/L)	0.867 (0.637 to 0.955)	<0.001
TP (g/L)	0.193 (-0.354 to 0.642)	0.490
ALB (g/L)	−0.066 (-0.560 to 0.462)	0.815
ALB/Urea (g/mmol)	−0.591 (-0.847 to −0.113)	0.020
Calcium (mmol/L)	0.038 (-0.484 to 0.540)	0.892
Magnesium (mmol/L)	−0.232 (-0.665 to 0.318)	0.405
Phosphate (mmol/L)	−0.412 (-0.763 to 0.127)	0.127
CHOL (mmol/L)	−0.067 (-0.560 to 0.461)	0.812
TRIG (mmol/L)	0.595 (0.119 to 0.849)	0.019
NEFA (mmol/L)	0.586 (0.105 to 0.844)	0.022
(NEFA + TRIG)/CHOL	0.617 (0.153 to 0.858)	0.014
BHB (mmol/L)	−0.129 (-0.602 to 0.410)	0.646
AST (U/L)	0.516 (0.006 to 0.813)	0.049
AST/BHB (U/mmol)	0.400 (-0.142 to 0.757)	0.140
AST/CHOL (U/mmol)	0.428 (-0.107 to 0.771)	0.111
GGT (U/L)	−0.277 (-0.691 to 0.274)	0.317
GLDH (U/L)	−0.091 (-0.577 to 0.442)	0.747
CK (U/L)	0.349 (-0.199 to 0.730)	0.203
LDH (U/L)	0.428 (-0.107 to 0.771)	0.111

## 4. Discussion

Our study brought the first data about the comprehensive changes in the serum proteome associated with the presence of RP in dairy cows. Using high-throughput TMT-based LC-MS/MS methodology, we identified a set of ten proteins with altered relative abundance in serum differing between the cows with RP and PP. The functions of these ten proteins, together with the direct relationship between the extent of the proteome changes and the intensity of APR, pointed out that a complex systemic response to RP designated the pattern of the proteome changes in serum.

Four of the ten proteins with the RP-associated abundance changes represented the archetypic positive acute phase proteins (APP) in ruminants: HP, LBP, ITIH 4, and fibrinogen. Accordingly, the observed serum proteome alterations, to a large extent, reflected the activation of the innate immune mechanisms (Ceciliani et al. 2012; Soler et al. 2019). Besides, the higher abundance of IGHM, a constituent of Immunoglobulin M (Saini and Kaushik 2001), evidenced the activation of the adaptive immunity pathways (Eckel and Ametaj 2016). Also, similarly to previous findings (Dervishi et al. 2016), the differences in SAA level between the studied groups indicated that the APR vigorously amplified in RP, when compared with low-grade systemic inflammation, commonly occurring in cows with no health issues after calving (Bradford et al. 2015). Further interpretation of our results allowed for hypothesizing about the amplification triggering mechanisms.

The increased uterine content of bacterial endotoxins, primarily lipopolysaccharide (LPS), has a well-established role as a risk factor for RP (Eckel and Ametaj 2016; Dervishi and Ametaj 2017, Boudelal et al. 2022). According to our results, the bloodstream flashover of LPS could induce a change in the serum proteome of the cows with RP. The protruding increase in the level of LBP confirms this feature, as this APP is primarily known for its ability to bind LPS and subsequentially elicit the APR (Ceciliani et al. 2012).

Despite the proinflammatory features of the stimulated bovine platelets (Weiss 1999), the current perception of RP pathophysiology has not satisfactorily appreciated their role in triggering the periparturient APR boost in RP. The results of our study are providing evidence of the enhanced platelet activation, degranulation, and aggregation in the cows with RP, all strengthened by the molecular responses to increasing cytosolic Ca^2+^ concentration. The proteome background of these mutual effects involved the increased abundance of FGA and ITIH4, jointly with the lowered CLEC3B abundance. The involvement of FGA is not surprising when considering that the adhesion of fibrinogen on the platelet surface represents a long-established trigger for their activation cascade (Frojmovic et al. 1996). During the physiological course of pregnancy in cows, FGA was reported to peak two weeks before parturition (Kurpińska et al. 2016). However, other authors (Heuwieser et al. 1990) detected a postpartum increase in fibrinogen concentration, whereby the levels were higher in cows with RP than in those with no difficulties in the placenta expulsion. Hence, the level of ITIH4 in dairy cows was known as a regulator of platelet degranulation and effects initiated by the increased Ca^2+^ concentration in their cytoplasm (Koch et al. 2021). The pathway enrichment in our study indicated that these adaptive mechanisms could transform into pathogenic in the environment of a strong APR, characteristic of RP. The decreased amount of TNCT in serum could point to the involvement of platelet hyperactivation in the extreme strengthening of APR in cows with RP. This hypothesis originated from the finding of Chen et al. (2020), explaining the reduction of TNCT blood levels in sepsis due to the binding to the high mobility group box-1 protein, whereby the appearing complexes induced pyroptosis and immunosuppression. Also, the lowered TNCT relative abundance could be a linkage with the impairments in plasminogen activation and endothelial functions (McDonald et al. 2020). Surprisingly, RP and PP groups in our study did not differ regarding FGA and TNCT concentrations as measured with the ELISA technique. These results might be due to the unequal proportions of FGA and TNCT isoforms with different biological and immunochemistry features, as described in human patients (Dardé et al. 2010; Shang et al. 2019).

Pathway enrichment analysis implied that the lowered abundance of serpin family D member 1 (SERPIND1), also known as heparin cofactor II, aggravated the abovementioned deleterious effects by causing a procoagulant hemostatic disbalance in cows with RP. Apart from anticoagulant function, studies on animal models revealed that SERPIND1 also showed vascular protective effects (Grover and Mackman 2022), implying that the lowered SERPIND1 level in cows with RP might also weaken the proper functioning of the endothelium. The circulus of thrombo-inflammatory mechanisms closed with pathways involving Apo A4. The peripartum fluctuations in Apo A4 level in healthy cows included peaking one month before calving, followed by a decrease with a nadir the first day after parturition, and increasing when cows entered lactation (Kurpińska et al. 2015). The lowered Apo A4 amount might negatively affect the activity of lipoprotein lipase and lipoprotein remodeling processes, thus consequentially enhancing endothelial dysfunction in cows with RP (Qu et al. 2019).

It is necessary to highlight that the RP-associated alterations of serum proteome involved the increased abundance of proteins with the protective biological role. Among them were HP and HPX, the traditional "allies against heme toxicity" (Smith and McCulloh 2015). Haptoglobin had the highest fold-change in RP versus PP group, and further validation results additionally confirmed the previously established link between RP occurrence and the increase in HP level (Pohl et al. 2015; Dervishi et al. 2016). In addition, the results obtained for HPX might establish a new positive bovine APP. Enrichment analysis identified heme scavenging from serum, together with the binding and uptake of ligands by scavenger receptors as the pathways in which HP and HPX participated. Very interestingly, the probability of intravascular hemolysis was negligible; instead of the decreased values, characteristic for hemolysis (Ceciliani et al. 2012), serum level of HP was increased, plus the activity of LDH was within the reference interval. Therefore, the immunomodulatory properties of HP and HPX came into focus, attributing the abundance increase in the cows with RP to the protective challenges of establishing control over the vigorous inflammation. The study of Bogado Pascottini et al. (2021) reinforced the importance of HP in controlling low-grade systemic inflammation, which is common in cows with no health issues after calving (Bradford et al. 2015). Namely, HP levels in healthy postpartum cows, along with a mild to moderate increase, showed a correlation with neutrophil activities: positive in relation to phagocytosis and proteolytical capacity; and negative related with oxidative burst. Although analogous studies for HPX are not available, the immunomodulatory features of HPX are clearly implied as it has hyaluronidase and serin protease activities, together with the capacity to diminish lymphocyte necrosis (Mauk et al. 2011), while human patients, who survived sepsis had higher HPX concentrations than the non-survivors (Smith and McCulloh 2015).

Higher GPX altered relative abundance in the RP group might be an additional protective mechanism, endeavouring to decrease oxidative stress associated with RP. However, the lack of difference in activity between the RP and PP groups questioned the protective efficiency. Opposing our findings, Li et al. (2021) detected 20% lower GPX activity in the plasma of cows with RP than in healthy cows. Interestingly, other authors considered the higher GPX activity in tissue of RP than in physiologically expelled placenta as part of the protective strengthening of the local antioxidant capacities (Kankofer et al. 2013). In our study, GPX inactivation in cows with RP by the increasing amount of reactive nitrogen species generated during the intensive APR might explain the disparity between GPX relative mass concentration and activity. The resulting accumulation of peroxide might have upregulated the expression of the GPX gene (Miyamoto et al. 2003).

The results of the studies addressing the tissue proteome differences between the retained and physiologically expelled bovine placenta (Fu et al. 2016, Wawrzykowski et al. 2019) did not include any of the ten proteins creating the serum profile of RP-associated proteome changes obtained in this study. That status was not surprising, as most of them were APP with the liver as the primary site of synthesis (Ceciliani et al. 2012). Nevertheless, the *vice versa* situation could be intriguing. A potential explanation could be that the changes in the tissue proteome indicated *in situ* mechanisms involved in RP pathogenesis and, as such, did not reach a significant amount in systemic circulation. Nevertheless, caution is necessary due to the methodology-related bias, as Wawrzykowski et al. (2019) used gel-base MS proteomic technique to analyze tissue proteome. Also, the semi-stochastic background of the DDA scanning approach (Michalski et al. 2011), which we used, allowed the possibility of omitting these placenta-derived proteins because they were not among the most abundant ones. In the clinical context, this issue holds relevance for explaining the absence of SAA among the proteomic results in our study. On the contrary, ELISA measurement performed independently during the laboratory evaluation revealed a 20 times higher level in the RP group than in the PP group, whereby clinical signs also indicated intensive inflammation in cows with RP.

Our study, analogously with previous reports (Dervishi et al. 2016), also evidenced that a vigorous APR accompanied RP. The increased cortisol level in the blood (Alhussien and Dang 2019) might cause higher glycemic values. The higher T-Bil concentration in RP was also noticed previously (Li et al. 2021), but might not have a substantial clinical relevance. The indexes combining the results of several routine laboratory tests may have supreme diagnostic performances when compared with the individual tests. The study of Mostafavi et al. (2013) showed better sensitivity and specificity of NEFA/CHOL than NEFA alone for diagnosing hepatic lipidosis. In our study, a whole set of ratios distinguished RP from the PP group, but the ROC analysis on our results marks their diagnostic accuracy as questionable. However, their screening potential for subtle metabolic alterations occurring in RP merits validation in larger cohorts.

Traditionally, the pathogenesis of RP relied on two features: inflammatory and metabolic, attributable to the disequilibria in glucose, lipid, and calcium metabolism. These features interact; nevertheless, understanding the hierarchy between them has been a matter of challenge (LeBlanc 2020; Mezzetti et al. 2020; Horst et al. 2021). In our study, the extent of RP-associated serum proteome changes correlated both with the intensity of APR and several indicators of metabolic disbalance. Nevertheless, multiple regression indicated only the positive relationship with the intensity of APR as independent. These results attached the proteomic changes to the batch of interrelated metabolic conditions (increased serum NEFA, hyperketonemia, and hypocalcemia), hierarchically shaped by the intensity of postpartum APR, thus reinforcing the leading role of the poorly controlled periparturient inflammation in the impaired health condition of dairy cows (Horst et al. 2021).

Serum concentrations of LBP, HP, and SAA differentiated between the RP and PP groups with acceptable sensitivity and specificity, thus marking these three APPs potential candidates for future studies on RP biomarkers. While thinking about their application in patients, several features appeared as challenging. The measurement for diagnostic purposes would not be rational due to the straightforwardness of the clinical findings in RP (Peter 2013; Sheldon 2018). Their predictive or prognostic role might be promising, as the data about peripartum fluctuations are already available for HP and SAA. The RP-related increase in HP level started during the fourth week before calving and was sustained until one month postpartum, while SAA was constantly increased during the same period (Dervishi et al. 2016). Chebel (2021) reported on prepartum HP level as a predictor of RP and metritis, thus providing the rationale for similar studies for the other two candidates. Finally, besides including much larger cohorts, the subsequent studies would also need to validate the current results on dairy cows of different breeds.

The small number of the cows studied marks our results as pilot. Also, all cows were multiparous and, therefore, less susceptible to the harmful effects of the periparturient inflammation (Pohl et al. 2015). In addition, the lack of detailed data about the accommodation and nutritional conditions (such as the ambient temperature, humidity, or the composition of the total mixed ration) did not allow the assessment of the eventual confounding effects of these variables. Finally, the successful recovery in all studied cows eliminated the opportunity of identifying the potential indicators of the risk for short-term mortality in the cows with RP.

## 5. Conclusion

The pattern of retained placenta-associated changes in the serum proteome of dairy cows, profiled using the high-throughput technique, consisted of alterations in the relative abundance of ten proteins. In the pathobiological context, the pattern recouped pathways regulating the innate immunity mechanisms with strengthening or ameliorating effects. The current findings offered a few candidates for prognostic biomarkers in cows with retained placenta, thus raising the necessity for further clinical validation studies.

## Supplementary Material

Supplemental Material
